# Effect of black tea extract on herpes simplex virus-1 infection of cultured cells

**DOI:** 10.1186/1472-6882-13-139

**Published:** 2013-06-18

**Authors:** Anthony Cantatore, Sade D Randall, Daniel Traum, Sandra D Adams

**Affiliations:** 1 Department of Biology and Molecular Biology, Montclair State University, Montclair, NJ 07043, USA

**Keywords:** Herpes simplex virus-1, Black tea extract, Theaflavins, Antiviral

## Abstract

**Background:**

The purpose of this investigation was to determine if black tea extract (BTE), consisting primarily of flavanol compounds called theaflavins, could inhibit herpes simplex virus type-1 (HSV-1) infection in cultured A549 (human epithelial) and Vero cells.

**Methods:**

The effect of BTE both on A549 and Vero cultured cells and on HSV-1 was assessed by using phase contrast and fluorescent microscopy, and cell viability and proliferation assays. After establishing the maximum non-cytotoxic concentration of BTE, A549 and Vero cells and HSV-1 virions were treated with varying concentrations of BTE, respectively. A549 and Vero cells were infected with HSV-1 with green fluorescent protein (GFP) insert at the UL46 gene. The effect of infectivity was determined by viral DNA extraction followed by PCR, plaque assays, adsorption assays, and electrophoresis of PCR products.

**Results:**

BTE was not cytotoxic to A549 and Vero cells, as confirmed by cell viability and proliferation assays, in which BTE treated groups paralleled the positive control group. For both cell lines, plaque assays and fluorescent microscopy indicated an inverse relationship between BTE concentration (from 0.14 μM – 1.4 mM) and HSV-1 infectivity. Specifically, PCR and electrophoresis showed a reduction in the viral genome following treatment with BTE. In addition, there was a noticeable decrease in the amount of viral plaques for BTE treated samples in the adsorption assays.

**Conclusions:**

BTE consisting primarily of theaflavins is not cytotoxic and can reduce or block the production of infectious HSV-1 virions in cultured A549 and Vero cells, thus inhibiting the infectivity of the virus by interfering in the attachment, penetration and viral DNA replication of HSV-1 particles. These findings indicate that BTE enriched with theaflavins has the potential to be developed as a safe, therapeutic antiviral agent to prevent the spread of HSV-1.

## Background

Herpes simplex virus type-1 (HSV-1) virions consist of an inner core with linear, double-stranded DNA that is enclosed in a capsid; an outer envelope containing various glycoproteins covers tegument proteins, which are exterior to the viral capsid [1,2].

The lytic infection cycle of HSV-1 begins with adsorption: when the virion first attaches to, then fuses with a host cell. Both attachment and penetration take place when viral glycoproteins bind to suitable receptors on the plasma membrane of the host cell. The presence of the receptors determines the tropism of HSV-1 and places a limit on the types of cells to which it is capable of attaching, and thus, infecting [3-5]. Green, oolong, and black teas are produced from the same plant, *Camellia sinensis*. While the main type of flavanol in green tea extract is the catechin, that in BTE is the theaflavin, a dimer of different catechins, which includes: theaflavin (TF-1), theaflavin-3-monogallate (TF-2A), theaflavin-3'-monogallate (TF-2B) and theaflavin-3,3'-digallate (TF-3) [6,7]. Since green tea is not fermented, it contains a relatively high amount of catechins as compared to black tea; fermentation causes the catechins to polymerize, which produces the theaflavins and thearubigens found in black tea, but absent in green tea [6].

The benefits of tea are often attributed to its antioxidant properties, which in turn have been ascribed to catechins, since green tea extract has been found to have more antioxidant activity than black tea extract [7]. However, recent studies have shown that concentrated theaflavin extracts made from black tea can be just as effective as catechins; it is believed that the high number of hydroxyl (OH) groups of theaflavins, which have been shown to protect cells against oxidative damage, is responsible for its antioxidative properties [8,9]. Additionally, it has been found that theaflavins are capable of inhibiting certain types of cancer [10], as well as inhibiting viruses, including bovine rotavirus and bovine coronavirus [11], HIV-1 [12,13], and three different subtypes of influenza strains [14].

While it has been shown that HSV-1 can be inhibited by compounds in green tea extract and a variety of other polyphenolic compounds [15], the purpose of this study was to determine if black tea extract with a concentrated amount of theaflavins (≥ 80%) (BTE) could also inhibit HSV-1. Since theaflavins in BTE are composed of a dimer structure formed from catechin monomers found in green tea, which have been found to inhibit HSV-1 [15], it is reasonable to infer that theaflavins in BTE may also produce similar results based on structural similarities. Despite the fact that theaflavin molecules are larger than catechins, larger polyphenolic compounds such as tannins have been shown to inhibit other viruses [16,17], indicating that the size of the molecule may not necessarily be a factor required for viral inhibition. Rather, the large amount of hydroxyl groups on these polyphenolic compounds seem to be the one common structural component among these various, natural viral inhibitors; thus, BTE, with high concentrations of theaflavins, may be an effective inhibitor of HSV-1.

## Methods

### Cells

Human epithelial (A549) cells [American Type Culture Collection (ATCC), Manassas, VA, USA] were cultured until confluent in 1X Ham's F-12K nutrient media, Kaighn's modification with 2 mM L-glutamine, supplemented to contain 10% fetal bovine serum (FBS) (Biowest, Miami, FL, USA) and 1 μg/mL gentamicin at 37°C and 5% CO_2_.

Vero cells [ATCC (Manassas, VA)] were cultured until confluent in Dulbecco Modified Eagle Medium (DMEM) with 5% FBS and 1μg/mL gentamicin at 37°C and 5% CO_2_.

### HSV-1 UL-46 virus maintenance

A recombinant strain of HSV-1, GHSV-UL46, which contains the sequence for green fluorescent protein (GFP) fused to the tegument protein pUL46, was used for all experiments [18] (ATCC, Manassas, VA, USA). Passage of virus was performed in T-25 flasks and cells were allowed to reach complete cytopathic effect (CPE). The media was then collected, centrifuged, and the supernatant containing virus kept in cryogenic vials at −80°C.

### Preparation of black Tea extract (BTE)

Black tea extract ≥ 80% theaflavins (BTE) (10 mg) (Sigma-Aldrich, Saint Louis, MO, USA) was dissolved in 1 mL of 10% FBS-media to produce a stock concentration of 14 mM BTE solution. Ten-fold dilutions (1.4 mM – 0.014 nM) of stock were stored in microcentrifuge tubes at 4°C.

### BTE cytotoxicity

#### Observation of cell morphology

Cell morphology was assessed using an Accu-Scope 3002 microscope by comparing treated and untreated samples. A549 and Vero cells were plated in 6-well plates, grown for 24 h, and then different concentrations of BTE (14 mM to 0.014 nM) were added to the wells. After 1 h the BTE was removed by aspiration and the cells were washed with PBS. Fresh media was added to the wells, and cells were examined at 400X for morphological changes after an additional 48 hour incubation at 37° and 5% CO_2_.

### Cell viability assays

A549 and Vero cells were plated in 6-well plates, and after 24 hours, different concentrations of BTE were added to each well. After one hour, the BTE was aspirated and the cells were washed with PBS, and cells, including control groups, were incubated with media for 24 hours at 37° and 5% CO_2_. Cells were then stained with trypan blue and counted using a hemocytometer.

### Cell proliferation assay

A549 and Vero cell suspensions (100 μL) were transferred to separate wells of a 96-well plate. To each well that contained a sample, 10 μL of cell proliferation reagent WST-1 (Roche Diagnostics, Indianapolis, IN, USA) was added; the plate was gently rocked, then placed in an incubator at 37°C and 5% CO_2_ for 30 minutes. The absorbance level for each well was measured at 450 nm in a microplate reader.

### Viral inhibition

#### Virus inactivation assay

100 μL of BTE solutions were mixed with 100 μL of HSV-1 in microcentrifuge tubes and incubated at 37°C and 5% CO_2_ for 1 hour. Then, 200 μL of each mixture was added to a separate well on a 6-well plate containing Vero cells, from which the media had been aspirated. The plates were incubated at 37°C and 5% CO_2_ for 1 hour and rocked every 15 minutes. After 1 hour, any unabsorbed virus was aspirated and 2.5 mL of 5% FBS-media was added to each well of Vero cells, and incubated at 37°C and 5% CO_2_ for 48 hours; then media from each well was harvested and used to infect fresh monolayers of Vero cells (70 - 80% confluent). Plates were incubated for 48 hours at 37°C and 5% CO_2_ and monitored for cytopathic effect. Virus titers were determined by plaque assays.

### Cell-treated extracts

A549 and Vero cells were plated in 6-well plates with 2.5 mL of cell suspension added to each well and incubated at 37°C and 5% CO_2_ until 80% confluent. The media was aspirated, and cells in each well were treated with 100 μL of one of the 10 concentrations of BTE solution. Plates were rocked and kept in an incubator at 37°C and 5% CO_2_ for 15 minutes. Unabsorbed solution was aspirated and 100 μL of virus was added to each well. The cells were incubated at 37°C and 5% CO_2_ for 1 hour and rocked every 15 minutes. After 1 hour, any unabsorbed virus was aspirated and 2.5 mL of 10% FBS-media was added to each well. The plates were incubated at 37°C and 5% CO_2_ for 48 hours, then media from each well was harvested and stored at −80°C.

### Virus-treated extracts

To obtain media with virions from Vero and A549 cells that had been infected with virus particles treated with BTE solutions, 100 μL of undiluted HSV-1 was mixed with 100 μL of BTE solution in a microcentrifuge tube for each of the 10 concentrations of BTE solution. The mixtures remained at room temperature for 15 minutes. Then, 200 μL of each mixture was added to a separate well on a 6-well plate containing A549 and Vero cells, respectively, from which the media had been aspirated. The plates were incubated at 37°C and 5% CO_2_ for 1 hour and rocked every 15 minutes. After 1 hour, any unabsorbed virus was aspirated and 2.5 mL of 10% FBS-media was added to each well of A549 cells, and incubated at 37°C and 5% CO_2_ for 48 hours, then media from each well was harvested and stored at −80°C.

### Viral titer determination using plaque assay

Ten-fold serial dilutions of cell-treated and virus-treated extracts of HSV-1 were prepared prior to infection. Confluent A549 and Vero cell monolayers were then infected with different dilutions of 100 μL HSV-1 and allowed to adsorb for 1 hour at 37 °C and 5% CO_2_. Unabsorbed viruses were aspirated, and plates were then overlaid with a nutrient medium-containing agar and incubated at 37°C and 5% CO_2_ for 3 days. Plaques were visualized by staining cells with crystal violet and counting within 50 hours. The plaque assay was carried out in triplicate.

### Plaque reduction assay

Experimental wells of 6-well plates containing confluent monolayers of A549 and Vero cells were infected with virus suspensions to produce 20–30 plaques per well. After 1 h incubation at 37 °C and 5% CO_2_, unabsorbed virions were aspirated. BTE solution (1.4 mM, 0.14 mM, 14 μM, and 0.14 μM, respectively) was then added to the appropriate wells, followed by nutrient medium-containing agar; the plates were incubated at 37°C and 5% CO_2_ for 3 days. Plaques were counted as described above.

### Virus adsorption assay

Equal volumes (100 μL) of BTE solution and a virus suspension, containing virus to yield 20–30 plaques per well, were placed in microcentrifuge tubes, and the mixtures were incubated at 37°C for 1 h. The samples were then placed on monolayers of A549 and Vero cells in 6-well plates and the virus was allowed to adsorb in the presence of the extract. Unabsorbed solutions were aspirated, and nutrient medium-containing agar was then added to each of the wells, and the plates were incubated at 37°C and 5% CO_2_ for 3 days. Adsorption efficiency was assessed by counting plaques, as described above.

### Virus attachment assay

BTE solution was added to wells of 6-well plates containing monolayers of A549 and Vero cells, and the plates were incubated at 4°C for 1 h. Extract solutions were then removed and virus suspensions containing virus to yield 20–30 plaques per well were added to each of the wells. Plates were incubated at 4°C for 2 h to allow attachment, then monolayers were rinsed 3 times with cold PBS; unabsorbed solutions were aspirated. Nutrient medium-containing agar was then added to each of the wells and the plates were incubated at 37°C and 5% CO_2_ for 3 days. Plaques were counted as described above.

### Virus penetration assay

Virus suspensions were prepared on ice to produce 20–30 plaques per well on monolayers of A549 and Vero cells in 6-well plates. Virus suspensions were placed on cells, and plates were incubated at 4°C for 2 h to allow attachment. BTE solution was then added to the wells at room temperature and plates were incubated at 37°C for 10 minutes to allow penetration. Unattached virions were then washed off with PBS (pH = 3), and unabsorbed solutions were aspirated. Nutrient medium-containing agar was then added to each of the wells and the plates were incubated at 37°C and 5% CO_2_ for 3 days. Plaques were counted as described above.

### Fluorescent microscopy

To visualize the effect that the BTE solution had on viral propagation, A549 and Vero cells were plated in 6-well plates. First, 100 μL of GHSV-UL46 was mixed with 100 μL of BTE solution in a microcentrifuge tube. The mixtures remained at room temperature for 15 minutes. Then, 200 μL of each mixture was added to a separate well on a 6-well plate that contained confluent cells. The cells were incubated at 37°C and 5% CO_2_ for 1 hour and rocked every 15 minutes. Any unabsorbed solution was aspirated from the cells and 2.5 mL of FBS-media was added to each well. The plates were incubated at 37°C and 5% CO_2_. Cells were observed with a fluorescent microscope, at 400X magnification every 6 hours post-infection for 24 hours.

### DNA extraction and quantification

DNA was extracted from infected A549 and Vero cells that contained either 10% FBS-media or 5% FBS-media, respectively or equal volumes HSV-1 virus treated in a microcentrifuge tube with either 1.4 mM BTE solution or 10% FBS-media (A549 cells), or one of the following HSV-1/BTE lysates: 0.14 mM, 14 μM, 1.4 μM, and 0.14 μM concentrations (Vero cells). Cells were incubated for 12 hours at 37°C and 5% CO_2_. The DNA from each of the five groups of cells was extracted with the Qiagen DNeasy® Blood & Tissue Kit (Qiagen Sciences, Germantown, MD, USA), following the manufacturer’s protocol. To quantify the total amount of DNA in both the extracted DNA and PCR products, a NanoDrop ND-1000 Spectrophotometer with accompanying computer software (NanoDrop Technologies Incorporated, Wilmington, DE, USA) was utilized, following the manufacturer’s protocol.

### Primer design and polymerase chain reaction (PCR) amplification of viral genes

Three sets of primers were designed to prime different regions of the HSV-1 genome based on published sequences: HSV-1 US6 (encoding glycoprotein D), HSV-1 GFP [19], and HSV-1 UL46 (encoding VP11/VP12) genes. The sequence, melting temperature (T_m_) and size of amplicons of forward and reverse primers are listed in Table 1. DNA (100 ng) extracted from treated HSV-1 infected Vero and A549 cells was added to each PCR reaction. Standard PCR amplification was performed in 25 μL reactions with an initial denaturation at 95°C for 2 minutes followed by 30 cycles of denaturation at 95°C for 30 seconds, annealing at 60°C for 1 minute and extension at 72°C for 30 seconds followed by a final extension period at 72°C for 10 minutes. Confirmation of the correct amplicon size was determined by 1% agarose gel electrophoresis and ethidium bromide staining.

**Table 1:** **The sequence, T**_**m **_**and amplicon size of the designed primers used in PCR**

**Primers**	**Target genes**	**Nucleotide sequence (5′ to 3′)**	**T**_**m **_**(°C)**	**Amplicon (nt)**
gD1F	HSV-1 US6	AGACGTCCGGAAACAACCCTACAA	64.6	752
gD1R		ACACAATTCCGCAAATGACCAGGG	64.6	
GFPF	HSV-1 GFP	TGACCCTGAAGTTCATCTGCACCA	64.6	717
GFPR		AACTCCAGCAGGACCATGTGAT	62.7	
VP1F	HSV-1 UL46	ACCAAGCCTTGATGCTCAACTCCA	64.6	957
VP1R		ACAACACGGTTCCCGAGAGTTTGA	64.6	

## Results

### Black tea extract concentrations up to 14 mM have no significant effect on cell morphology

A549 and Vero cells were exposed to ten-fold dilutions of BTE, from 14 mM to 0.014 nM. No significant changes in morphology, as determined by phase contrast microscopy, were observed at any tested concentration of BTE in A549 cells (data not shown). However, slight changes in morphology were observed for Vero cells at the highest concentration (data not shown). Vero cells appeared to tolerate 1-hour exposure to BTE up to 1.4 mM (data not shown).

### BTE does not reduce cell viability

**Figure 1:**
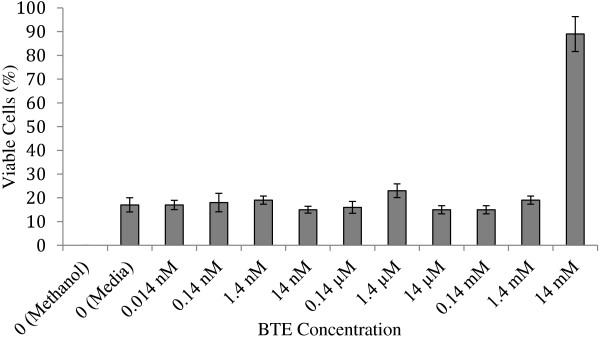
**Trypan blue assay results for BTE cytotoxicity in A549 cells.** Results indicate the percentage of live or viable cells as compared to the total number of cells for each sample (an average of three separate samples) (data not shown). Values represent ± SD.

The cell viability was quantitatively determined by using trypan blue and hemocytometer direct cell count to detect the effect of BTE on A549 cells (Figure 1). The viability of the BTE-treated cells was similar to the positive control group treated with 10% FBS-media. As the concentration of BTE increased, the percentage of cell death did not increase. The tested concentrations of BTE, from 14 mM to 0.014 nM, did not appear to be cytotoxic to A549 cells. One unexplained deviation from the group was the 14 mM BTE, which had a significantly higher percentage of live cells compared to any other group; these results were comparable for Vero cells (data not shown). This BTE concentration, therefore, was not used in the inhibition studies.

### Cell proliferation and viability assay indicates that BTE is not toxic to A549 and Vero cells

**Figure 2:**
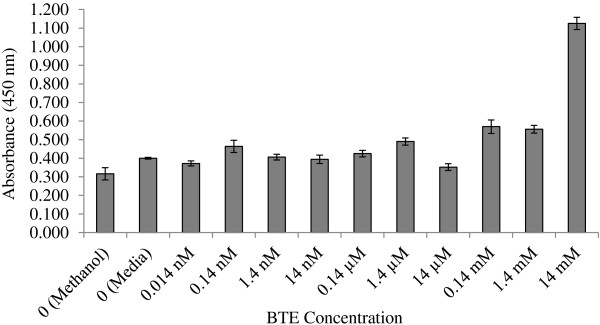
**WST-1 assay results for BTE cytotoxicity in A549 cells.** Results indicate the absorbance level (an average of three separate samples on the same plate), which relates to the amount of live or viable cells for each treated sample. A higher absorbance level indicates more living or viable cells than a lower absorbance level. As a reference, media containing no live cells with WST-1 produced an absorbance level of 0.211 (data not shown). Values represent ± SD.

To confirm the findings established by the trypan blue assay, an assay using WST-1 reagent was conducted. In this assay, only live cells can reduce WST-1, which is light red, to formazan, which is dark red; thus, the higher absorbance level is indicated by a darker color, which correlates to the number of living cells. Overall, the findings with the WST-1 assay for both A549 cells (Figure 2) and Vero cells (data not shown) paralleled those found for the trypan blue assay.

### Black tea extracts reduce HSV-1 viral titers

To visually observe the cytopathic effect (CPE) that HSV-1 had on A549 and Vero cells and to determine if BTE could inhibit HSV-1, either by reducing or preventing the observable CPE, treated and untreated cells infected with HSV-1 were observed at 400X magnification using phase contrast microscopy. Clear differences between each group were seen 12 hours and 24 hours post-infection (data not shown).

Plaque assays were conducted to test the effect of BTE on HSV-1. Titers determined by plaque assays of viral extracts in A549 and Vero cells are reported in Table 2. Treatment with BTE resulted in significantly reduced viral titers, as compared to untreated groups. Treatment of virions with various concentrations of BTE for one hour resulted in significantly reduced viral titers, as compared to untreated virus.

**Table 2:** Viral titers obtained from HSV-1 infected cultured cells treated with different BTE concentrations

**BTE Concentration**	**Viral Titer (PFU/mL)**
0 mM	8.0 x 10^6 a^
	2.5 x 10^6 b^
0.14 μM	4.6 x 10^5 c^
1.4 μM	3.4 x 10^5 b^
	6.0 x 10^4 c^
14 μM	3.0 x 10^5 b^
	1.6 x 10^4 c^
1.4 mM	0 ^a^
	2.2 x 10^2 c^
14 mM	0 ^a, c^

^a^ A549 cells ^b^ Vero cells ^c^ 1 hr treatment

### Fluorescent microscopy confirms the effectiveness of BTE in inhibiting HSV-1 propagation

**Figure 3:**
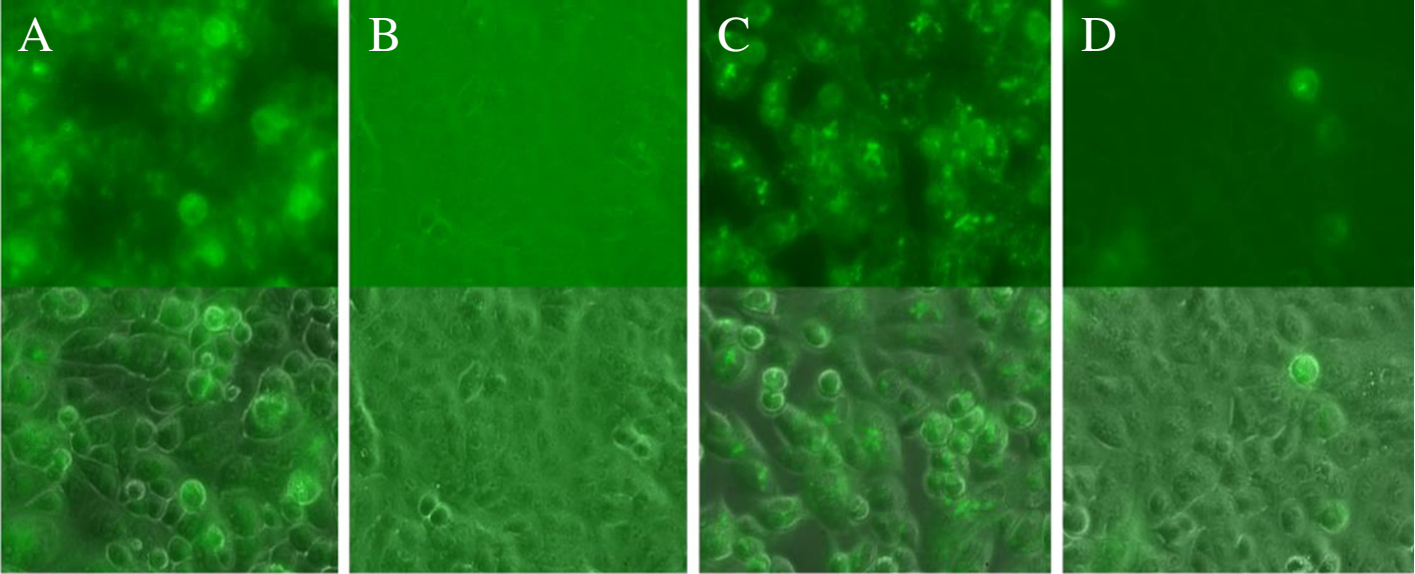
**Microscopy (400X magnification) of A549 cells.** Panel A: A549 cells inoculated with HSV-1 taken at 12 hours post-infection; Panel B: A549 cells infected with HSV-1 treated with 1.4 mM BTE, taken 12 hours post-infection. Panel C: A549 cells inoculated with HSV-1, taken 24 hours post-infection. Panel D: A549 cells infected with HSV-1 treated with 1.4 mM BTE, taken 24 hours post-infection. Upper panels: fluorescent image; Lower panels: merged image of fluorescent and phase contrast images.

**Figure 4:**
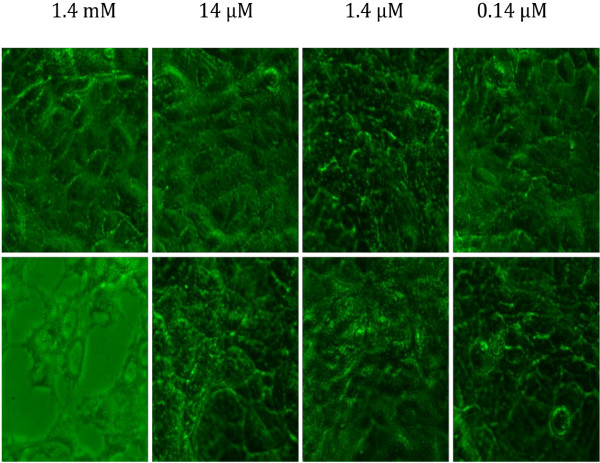
**Fluorescent Microscopy (400X magnification) of Vero cells infected with HSV-1 taken 36 h post-infection.** Upper panel: HSV-1 treated with 1.4 mM to 0.14 μM concentrations of BTE**;** Lower panel: Untreated HSV-1.

To confirm the findings of phase contrast microcopy and the plaque assay, fluorescent microscopy (400×) was employed to visually examine progeny virions in cells that were exposed to HSV-1 treated with 1.4 mM of BTE. For A549 samples, at 12 hours post-infection, there was a pronounced fluorescence from cells infected with untreated HSV-1 (Figure 3A), yet no viral fluorescence was detected from either the control (cells treated with 10% FBS-media) (data not shown) or cells inoculated with HSV-1 treated with BTE (Figure 3B). At 24 hours post-infection, there was still a significant amount of fluorescence from cells infected with untreated HSV-1 (Figure 3C), but only a small amount of fluorescence from cells inoculated with HSV-1 treated with BTE (Figure 3D). For Vero cells infected with untreated HSV-1, there was a significant amount of fluorescence 36 hours post-infection; Vero cells infected with increasingly higher concentrations of BTE showed decreasing levels of fluorescence (Figure 4).

PCR amplification of BTE-treated HSV-1 infected A549 and Vero cells indicates that the replication of viral genes for glycoprotein D, GFP, and VP11/12 is reduced following treatment of HSV-1 with higher concentrations of BTE.

**Figure 5:**
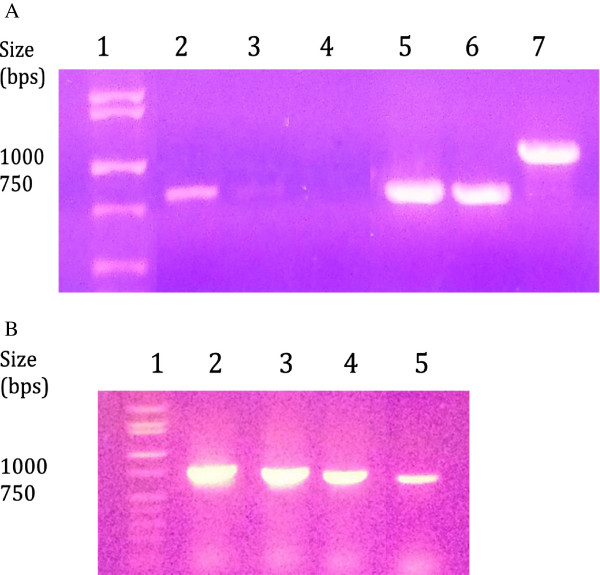
**Gel electrophoresis of PCR products. A**. PCR products extracted from HSV-1 infected A549 cells either treated with 1.4 mM BTE (columns 2–4) or untreated (columns 5–7). Column 1 contains the DNA ladder, with visible bands identified to the left in base pairs (bp). Columns 2 and 5, 3 and 6, 4 and 7 contain DNA amplified with primers for the HSV-1 gD, GFP and pUL46 genes, respectively. **B***.* Gel electrophoresis of HSV-1 GFP PCR products extracted from HSV-1 infected Vero cells either untreated (column 2) or treated with 0.14 μM, 1.4 μM, or 1.4 mM BTE (columns 3 – 5, respectively). Column 1 contains the DNA ladder, with visible bands identified to the left in base pairs (bp).

To determine if treatment with BTE interfered with the production of viral genomes, PCR was used to compare the relative levels of total DNA produced by infection with BTE-treated and untreated HSV-1. There was approximately a 75% reduction in the concentration of DNA in cells following treatment with 1.4 mM BTE (Table 3). Gel electrophoresis of the PCR products from DNA (extracted from HSV-1 infected A549 cells) resulted in visible bands on the gel corresponding to viral genes for glycoprotein D (gD), GFP and pUL46, apparent for untreated HSV-1 and HSV-1 treated with 1.4 mM BTE; however, the former had a higher intensity than the latter (Figure 5A). Sequence-specific primers were also used to amplify the viral DNA (extracted from HSV-1 infected Vero cells) encoding viral GFP at 12 hours post-infection for untreated HSV-1 or HSV-1 treated with varying concentrations of BTE (Figure 5B). The intensity of viral DNA products obtained after infection with untreated HSV-1 (column 2), was greater than that of HSV-1 treated with 0.14 μM, 1.4 μM, or 0.14 mM BTE (columns 3 – 5). Subsequent experiments focused on how higher concentrations of BTE affected HSV-1 infectivity.

**Table 3:** Quantification of DNA from Infected A549 Cells

**A549 Sample**	**Total DNA (ng/μL)**
Mock-Infected	240.7 ± 10.8
Untreated HSV-1	391.7 ± 4.0
BTE Treated HSV-1	278.2 ± 10.2

Values represent ± SD of the average of three samples (data not shown). The concentration of BTE was 1.4 mM.

### BTE inhibited viral adsorption in A549 and Vero cells through the combined effects of preventing viral attachment and penetration

**Figure 6:**
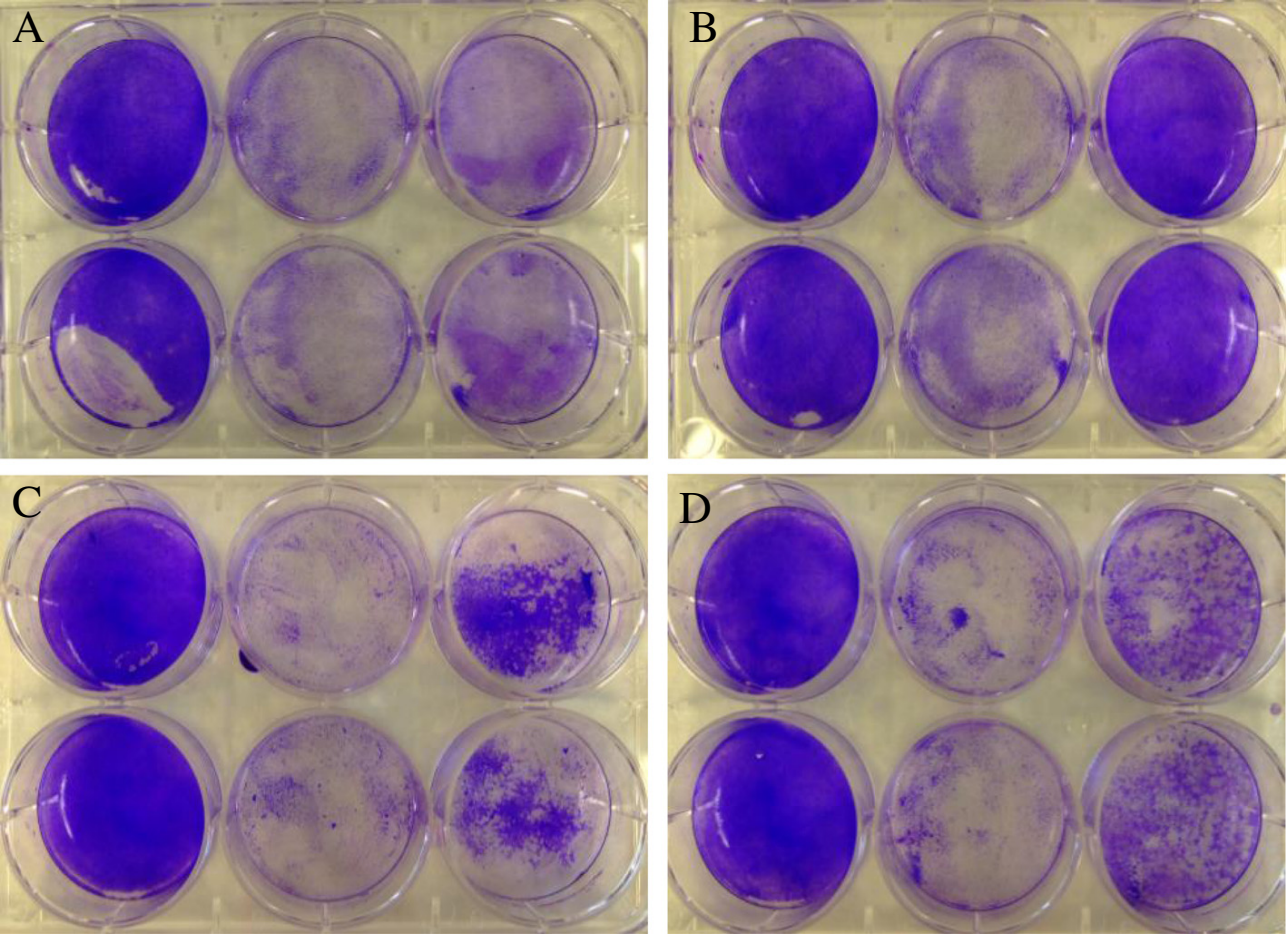
**Viral titer determination using plaque assays. A.** Plaque reduction assay results showing (from left to right wells): A549 cells exposed to media only, A549 cells exposed to HSV-1 and media and A549 cells exposed to HSV-1 and BTE. A549 cells were exposed to HSV-1 before being treated with BTE. **B**. Virus adsorption assay results showing (from left to right wells): A549 cells exposed to media only, A549 cells exposed to HSV-1 and media and A549 cells exposed to HSV-1 and BTE. A549 cells were exposed to HSV-1 treated with BTE. **C***.* Virus attachment assay results showing (from left to right wells): A549 cells exposed to media only, A549 cells exposed to HSV-1 and media and A549 cells exposed to HSV-1 and BTE. A549 cells were treated with BTE before being exposed to HSV-1. **D**. Virus penetration assay results showing (from left to right wells): A549 cells exposed to media only, A549 cells exposed to HSV-1 and media and A549 cells exposed to HSV-1 and BTE. A549 cells were exposed to HSV-1 to allow attachment, but not penetration; cells were subsequently treated with BTE, then the virus was allowed to penetrate the cells.

To determine if treatment with BTE interfered with viral adsorption in A549 and Vero cells, either in part or in whole, four assays were performed and compared to an untreated sample infected by HSV-1. In the plaque reduction assay a slight reduction in plaques was observed in the BTE treated group (Figure 6A), possibly due to the inhibitory effects of BTE previously mentioned. The virus adsorption assay (Figure 6B) showed a reduced number of plaques in the BTE treated sample, indicating that some part of adsorption was affected. Two additional assays were performed to further assess which aspect of viral adsorption was affected. The virus attachment assay displayed a significant reduction in the plaques formed in the BTE treated group (Figure 6C), while the penetration assay showed a similar reduction in plaque formation in the BTE treated group (Figure 6D).

## Discussion

The leading consumed beverage worldwide, after water, is tea. For over 50 centuries, tea has been recognized for its medicinal uses as an herbal treatment of multiple ailments that range from simple indigestion to atherosclerosis [7,19]. Theaflavins, polyphenols found mainly in black teas, are natural antioxidants and viral inhibitors. As such, black tea extracts may be useful in future pharmaceutical developments [20].

One concern for human health is HSV-1. Infections caused by this herpesvirus are estimated to affect anywhere from 45% - 98% of the world population, and up to 40% of these infected individuals are subject to recurrent outbreaks that most often result in infectious lesions and ulcerations of the skin. While treatments, such as the drug acyclovir, are in use today, most rely on the presence of a viral protein, thymidine kinase, to inhibit viral replication; mutant viral strains lacking this enzyme are still infectious but do not respond to the available medications. In addition, current treatments can have detrimental side effects and often require frequent doses that can be expensive [16,21,22]. Thus, alternative, lower cost treatments to HSV-1 infections are necessary to alleviate the symptoms of infected individuals.

Black tea extracts have previously been found to block the production of free radicals and inhibit the growth of cancerous cells, as well as exhibit cytotoxic effects against immortalized cells [23]. The purpose of this study was to assess the antiviral qualities of a black tea extract and determine its lowest inhibitory concentration against HSV-1. This hypothesis stems from the findings that black tea compounds have been shown to inhibit some viruses [11-14]. In addition, a green tea catechin, EGCG, has already been shown to inhibit HSV-1 [15,24]; it is suggested that this compound binds to glycoproteins on the envelope of the virus, thereby preventing viral entry into the host cell [24]. Since black tea theaflavins are merely polymers of green tea catechins [6], it is possible that the former may also inhibit HSV-1, though through a different mechanism. In addition, the flavanols in black tea may be more stable than those in green tea. Although the stability of green tea catechins is pH dependent, EGCG and EGC were less stable than EC and ECG, regardless of pH [25]. Theaflavins, however, were reported to be more stable at pH 7 than EGC and EGCG [26]. The increased stability of theaflavins at neutral pH could make these black tea compounds a more feasible option for the design of an antiviral therapeutic agent than EGCG.

Inhibition was measured visually, through observations that utilized both phase contrast and fluorescent microscopy, as well as quantitatively, by determining viral titers with the plaque assay method and viral DNA concentrations with samples extracted from infected cells. Phase contrast microscopy and plaque assays demonstrated that BTE significantly inhibited the infectious cycle of HSV-1, consistent with findings of previous studies [11-14]. These experiments demonstrated that non-cytotoxic concentrations of BTE can effectively inhibit the infectious cycle of HSV-1 in cultured cells. Similarly, treatment with BTE for one hour significantly reduced viral titers but did not inactivate the virions. Fluorescent microscopy revealed that treatment of HSV-1 virions with higher concentrations of BTE interfered with the infectious cycle of the virus in cultured A549 and Vero cells. Specifically, PCR and gel electrophoresis indicated that higher concentrations of viral DNA are produced in untreated HSV-1 infections, as compared to lower viral DNA concentrations from BTE-treated HSV-1. Also, a direct relationship between the increased BTE concentration and reduced intensity of samples containing viral GFP suggests that there is a significant reduction in viral genome replication in BTE-treated HSV-1 infected A549 and Vero cell cultures. Additional plaque assays indicated that both the attachment and penetration processes of HSV-1 adsorption in A549 cells and Vero cells are inhibited by BTE concentrations of 1.4 mM and 14 μM. Experimental results taken a whole indicate that BTE at non-cytotoxic concentrations can inhibit viral propagation by limiting the viral processes of replication and adsorption.

It has been reported that treatment of HSV-1 with TF-3 for 1 h completely inactivated the virus [27]. The effect of treatment of HSV-1 with BTE for 1 h was dose dependent. Our results indicate that the virus is not inactivated following 1 h treatment with BTE; therefore, the action of TF-3 alone may not explain the efficacy of BTE. Treatment with 1.4 mM BTE caused a reduction in the amount of HSV-1 genome synthesized 12 h after infection at this concentration and a lower viral count. BTE has been reported to lack cytotoxic effects on cultured cells, consistent with our findings [10]. Therefore, BTE concentrations up to 1.4 mM can be used to treat cells infected by HSV-1 and study its inhibitory effects. Data presented here indicate that BTE can be safely applied to cells at the concentrations tested. The lower cost of BTE, as compared to pure theaflavins, make it attractive for consideration as a safe and effective as an antiviral agent.

## Conclusions

BTE, consisting primarily of theaflavins, at concentrations of 0.14 μM and higher reduce or block the production of infectious HSV-1 virions in cultured A549 and Vero cells, thus inhibiting the infectivity of the virus by interfering in the attachment and penetration, as well as the DNA replication of HSV-1 particles. These findings indicate that BTE enriched with theaflavins has the potential to be developed as a safe therapeutic antiviral agent to prevent the spread of HSV-1.

## Competing interests

The authors declare that they have no competing interests.

## Authors’ contributions

SDA and AC designed the study. SDA supervised AC, SDR, and DT in the laboratory. SDA and AC drafted the manuscript. AC conducted all experiments using A549 cells. SDA, SDR and DT conducted experiments using Vero cells. All authors read and approved the final manuscript.

## Pre-publication history

The pre-publication history for this paper can be accessed here:

http://www.biomedcentral.com/1472-6882/13/139/prepub
